# Effects of proton versus photon irradiation on (lymph)angiogenic, inflammatory, proliferative and anti-tumor immune responses in head and neck squamous cell carcinoma

**DOI:** 10.1038/oncsis.2017.56

**Published:** 2017-07-03

**Authors:** M Lupu-Plesu, A Claren, S Martial, P-D N'Diaye, K Lebrigand, N Pons, D Ambrosetti, I Peyrottes, J Feuillade, J Hérault, M Dufies, J Doyen, G Pagès

**Affiliations:** 1 University of Nice Sophia Antipolis, Nice, France; 2 Institute for Research on Cancer and Aging, Nice, CNRS UMR 7284, INSERM U1081, Nice, France; 3 University of Aix Marseille, Marseille, France; 4 Centre Antoine Lacassagne, Nice, France; 5 University of the Côte d'Azur, CNRS, Institute of Molecular and Cellular Pharmacology, Sophia Antipolis, France; 6 Nice University Hospital, Nice, France

## Abstract

The proximity of organs at risk makes the treatment of head and neck squamous cell carcinoma (HNSCC) challenging by standard radiotherapy. The higher precision in tumor targeting of proton (P) therapy could promote it as the treatment of choice for HNSCC. Besides the physical advantage in dose deposition, few is known about the biological impact of P versus photons (X) in this setting. To investigate the comparative biological effects of P versus X radiation in HNSCC cells, we assessed the relative biological effectiveness (RBE), viability, proliferation and mRNA levels for genes involved in (lymph)angiogenesis, inflammation, proliferation and anti-tumor immunity. These parameters, particularly VEGF-C protein levels and regulations, were documented in freshly irradiated and/or long-term surviving cells receiving low/high-dose, single (SI)/multiple (MI) irradiations with P/X. The RBE was found to be 1.1 Key (lymph)angiogenesis and inflammation genes were downregulated (except for *vegf-c*) after P and upregulated after X irradiation in MI surviving cells, demonstrating a more favorable profile after P irradiation. Both irradiation types stimulated *vegf-c* promoter activity in a NF-κB-dependent transcriptional regulation manner, but at a lesser extent after P, as compared to X irradiation, which correlated with mRNA and protein levels. The cells surviving to MI by P or X generated tumors with higher volume, anarchic architecture and increased density of blood vessels. Increased lymphangiogenesis and a transcriptomic analysis in favor of a more aggressive phenotype were observed in tumors generated with X-irradiated cells. Increased detection of lymphatic vessels in relapsed tumors from patients receiving X radiotherapy was consistent with these findings. This study provides new data about the biological advantage of P, as compared to X irradiation. In addition to its physical advantage in dose deposition, P irradiation may help to improve treatment approaches for HNSCC.

## Introduction

Approximately 50% of all cancer patients are subject to radiotherapy during the course of their illness with an estimation that radiotherapy contributes to approximately 40% towards curative treatment.^1^ The goal of radiotherapy is to deliver loco-regionally a specific dose of radioactivity that will allow the destruction of cancer cells, while limiting the exposure of surrounding healthy tissues. Among the ionizing radiation treatments, the large majority consists of photons (X) of high energy (5–20 MeV). However, the main disadvantage of X radiotherapy is represented by the deposition of radiation also at the level of surrounding healthy tissues, leading to side effects. Although the ionizing radiation by proton beams (P) is currently more expensive and more difficult to produce, it has the physical advantage of delivering no radiation outside of the intended targeted area, thanks to the so-called Bragg peak.^2^

P radiotherapy is mainly proposed for the treatment of uveal melanoma, skull base and paraspinal tumors due to its high precision in tumor targeting with a very high irradiation dose next to radiosensitive structures.^2^ It is also proposed for the pediatric tumors based on the advantage to deliver a much lower integral dose, which significantly reduces the risk of radiation induced cancers in a long-life expectancy setting.^2^ Several retrospective and dosimetry studies have suggested an advantage of P radiotherapy in other tumors located near organs at risk, such as the head and neck squamous cell carcinoma (HNSCC).^2^

The head and neck cancers are among the 10 most common types of cancer and the seventh cause of mortality from cancer worldwide. Depending on disease stage, the treatment of HNSCC consists of either chemoradiotherapy and/or surgical excision.^3^ However, conventional radiotherapy with X in HNSCC remains difficult, due to the proximity of numerous organs at risk (that is, salivary glands, esophagus and larynx). Recent studies have shown an advantage of P, over X radiotherapy, in inducing lower toxicities^4^ and lower dose delivery to organs of risk^5^ in HNSCC patients.

Despite of the currently available therapeutic strategies, the five-year overall survival rate of HNSCC patients is only 53%^6^ because of a high percentage of a poor response to therapy and high recurrence rates. Sentinel lymph node metastasis, the first sign of tumor progression, was directly correlated to prognosis in HNSCC patients.^7^ Vascular endothelial growth factor C (VEGF-C) is a major pro-lymphangiogenic factor responsible for the metastatic dissemination of cancer cells.^8^ A significant correlation has been observed between intra-tumor lymphatic vessel density and lymph node metastasis in patients with HNSCC.^9^ Moreover, VEGF-C expression levels correlated with lymphatic vessel density and lymph node metastasis in these patients.^10, 11^ VEGF-C-dependent development of the lymphatic network might also be the major route of spread of tumor cells when the patients become resistant to therapy.^8^

Beside the physical advantage of P versus X irradiation and the RBE, few comparative preclinical studies have been conducted that contrast cellular/biological response to P versus X radiations.^12, 13, 14, 15^

P irradiation led to distinct gene and protein expression profiles.^12^ Mice receiving total-body irradiation with either P or X had enhanced plasma levels of transforming growth factor-β, only after X irradiation.^13^ Moreover, X irradiation promoted angiogenesis, thus enhancing metastasis by upregulation of various pro-angiogenic factors.^14^ By contrast, low dose P irradiation did not induce the pro-angiogenic and pro-inflammatory genes, impaired tumor cell invasion *in vitro* and attenuated tumor growth rate in mice.^14^ By downregulating integrins and matrix-metalloproteinases (MMP), P irradiation also reduced invasive and migratory properties of tumor cells.^15^

Therefore, beside the physical advantage in dose deposition, P may have different biological properties, as compared to X radiation at a similar dose. The purpose of the present study was thus to analyze the different biological behaviors of HNSCC cells when exposed to P versus X radiation. The study focused on molecules with key roles in the progression and prognosis of HNSCC, such as the inflammatory cytokines: Interleukin 6 (IL6),^16^ Interleukin 8 (IL8);^17^ (lymph)angiogenic factors: VEGF A, C and D and their receptors: vascular endothelial growth factor receptor (VEGFR) 1, 2 and 3, Neuropilin (NRP) 1 and 2;^18, 19^ factors involved in lymphatic vessels development: lymphatic vessel endothelial hyaluronan receptor 1 (LYVE1), prospero homeobox 1 (PROX1) transcription factor, and podoplanin (PDPN), a mucin-type transmembrane protein^20^; pro-inflammatory chemokine C-C Motif Chemokine Ligand 2 (CCL2) involved in cell migration^21^; cell cycle regulators: polo-like kinase 1 (PLK1)^22^ and telomeric repeat-binding factor 2 (TRF2) transcription factor^23^; immune checkpoint molecule programmed death-ligand 1 (PD-L1) involved in anergy and tumor progression.^24^

The role of the above-mentioned molecules in the post-irradiation progression of HNSCC has not been elucidated. Our working hypothesis was that different radiation types would lead to different intrinsic and extrinsic biological responses, allowing the adaptation of tumor cells. Therefore, we studied the impacts of P versus X irradiation on human HNSCC cells viability; proliferation; whole transcriptome profile and expression of key genes/proteins implicated in (lymph)angiogenesis/metastasis, inflammation, tumor cell proliferation and anti-tumor immunity; tumorigenic potential, and depicted the molecular mechanisms of post-irradiation VEGF-C regulation, to set the basis for improved therapeutic approaches for HNSCC.

## Results

### Cell survival/proliferation is in favor of P following single irradiation, and X following multiple irradiations

Our hypothesis was that irradiation would lead to different cell viability and proliferation profiles depending on the radiation type and dose, number of irradiations and time of assessment. We qualified as the ‘acute response (AR)’ the modifications of biological parameters (proliferation, survival, gene expression) a few hours following a single irradiation (SI). The modifications of the same biological parameters on cells that have survived to multiple irradiations (MI) and that have been expanded as new populations were qualified as the ‘chronic response (CR)’.

In order to calibrate our experiments, we first determined a relative biological effectiveness (RBE) of photons and protons on our model cell lines following SI. According to the literature, P therapy treatments are based on a RBE of 1.1, relative to high-energy X therapy.^25^ The surviving curve of CAL33 cells following administration of escalating doses of either P or X irradiation confirmed a RBE of 1.1 for P, as compared to X irradiation (Supplementary Figure S1). This experiment confirms the literature data showing that P kills tumor cells more efficiently than X irradiation.^25^

**Figure 1:**
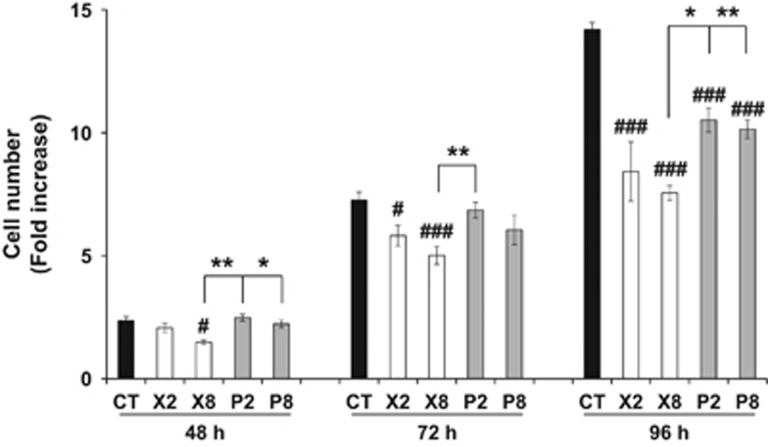
CAL33 proliferative ability following multiple X or P irradiations. Counts of CAL33 cells following multiple low (2 Gy) or high (8 Gy) dose(s) of P or X irradiation and cell expansion after the third irradiation (CR-MI). The values correspond to fold increase, as compared to the viable cell number at 24 h after cell seeding. Significantly decreased viable cell counts, as compared to CT: #, *P*<0.05; ###, *P*<0.001. Significantly increased viable cell counts for comparisons between X and P groups: *, *P*<0.05; **, *P*<0.01. CT, control (non-irradiated cells).

However, patients are irradiated several times to reach a maximal therapeutic efficacy. Therefore, our next purpose was to compare the relative aggressiveness of cells that were resistant to MI by X or P. Hence, we performed our experiments on two independent cell lines (CAL33 and CAL27). The proliferative ability along a time course of CAL33 (Figure 1) or CAL27 (Supplementary Figure S2) that have survived to MI was determined. As compared to non-irradiated cells, the proliferation of X or P irradiated cells was reduced in both models and the difference was striking 96 h following cell seeding (*P*<0.001 for CAL33; *P*=0.049 for CAL27). However, the difference in proliferation became statistically significant earlier for X irradiated cells in the CAL33 model (*P*=0.02 for X8 at 48 h; *P*=0.014 and 0.009 for X2 and X8, respectively, at 72 h; *P*<0.001 for all conditions at 96 h). Whereas the difference in proliferation did not reach statistical significance between X2 and X8 irradiated cells, X8 cells proliferated to a lesser extent, as compared to P2 and P8, at 48 h post seeding (*P*=0.006 and *P*=0.035, respectively), to P2 at 72 h post seeding (*P*=0.018), and P2 and P8 irradiated cells at 96 h post seeding (*P*=0.012 and *P*=0.008, respectively).

Therefore, the overall therapeutic advantage, attested by reduced cell viability and proliferation capacity following SI switched in favor of X post MI for CAL33 cells. For CAL27, no difference in the proliferative ability of MI X and P cells was observed, suggesting that X and P exert different outcomes, depending on the HNSCC type.

### P irradiation leads to overall lower induction of mRNA coding pro-inflammatory, pro-(lymph)angiogenic and pro-proliferative genes

The gene expression levels for CAL33 cells following SI or MI, represented as percentage of control, and the gene expression scores are listed in Table 1a and b. The mRNA levels of the different tested genes overall increased in a dose-dependent manner and with the irradiation number after both P and X irradiation. Genes involved in (lymph)angiogenesis, inflammation and immune tolerance were overall less expressed after high dose(s) of P, as compared to X, irradiation in all investigated groups; the genes involved in (lymph)angiogenesis, inflammation and immune tolerance were downregulated after P irradiation, showing significantly lower mRNA levels, as compared to X irradiation, within the following settings: (i) AR-SI after low dose: CCL2 (*P*=0.035) and high dose: IL-6 (*P*=0.0001); (ii) CR-MI after low dose: VEGF-A (*P*<0.0001), IL-6 (*P*<0.0001), IL-8 (*P*=0.046), CCL2 (*P*=0.041), PD-L1 (*P*=0.002) and high dose: VEGF-D (*P*<0.0001) and IL-8 (*P*<0.0001). By contrast, among these genes, X irradiation led to downregulation of IL-8 only, within the low dose CR-MI settings. Notably, VEGF-C mRNA levels were systematically increased after both P and X irradiation, but they were significantly lower after P, as compared to X irradiation, after high dose within the CR-MI setting (*p*<0.001). Among all investigated genes, IL-8 was the gene whose mRNA was induced at the highest level after X (79-fold, as compared to control), but not after P irradiation, within the high dose CR-MI setting (*P*<0.0001). Moreover, both P and X irradiation augmented PD-L1 mRNA expression in a dose-dependent manner within the AR-SI and CR-MI settings, and in an irradiation number-dependent manner within the AR-SI setting. The generated gene expression scores showed that P irradiation is associated with a more favorable profile (reduced proliferation, (lymph)angiogenesis, inflammation)). A similar gene score, in favor of P irradiation, was also obtained for CAL27 cells, within the CR-MI setting, despite an increase in VEGF-C, VEGF-D, NRP1, NRP2, IL-8 and PD-L1 mRNA expression (Supplementary Table S3).

### Induction of VEGF-C protein is reduced in P irradiated cells

Because lymph node metastasis is frequent at diagnosis in HNSCC and in patients who relapse locally after radiotherapy, we focused our research on VEGF-C, the major growth factor for lymphatic endothelial cells. Although the mRNA levels of VEGF-C were increased after both low and high dose(s) of P or X irradiation, they were lower after high dose(s) of P irradiation. To confirm the results obtained at mRNA level, we next assessed VEGF-C protein levels in CAL33 and CAL27 cells.

**Figure 2:**
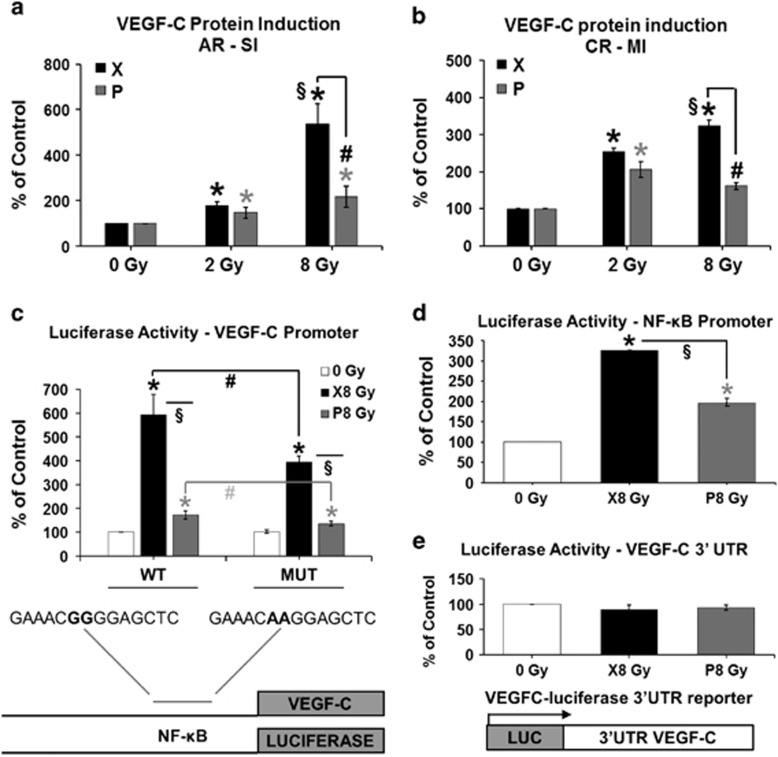
VEGF-C protein expression levels and regulation in CAL33 cells following P or X irradiation. (**a**) VEGF-C protein levels at 48 h post-single irradiation (AR-SI): * and *, significantly (*P*<0.05) increased levels after a low (2 Gy) or high (8 Gy) dose of P and X irradiation, respectively, as compared to CT; **#**, significantly decreased levels after a high dose of P, as compared to X irradiation; **§**, significantly increased levels after a high, as compared to a low X irradiation dose; (**b**) VEGF-C protein levels after cell expansion following the third irradiation (CR-MI): * and *, significantly increased levels after low and high doses of P and X irradiation, respectively, as compared to CT; Concentration in ng/ml, normalized to 1 × 10^6^ cells, and represented as percentage of CT. **#**, significantly decreased levels after high doses of P, as compared to X irradiation; **§**, significantly increased levels after high, as compared to low doses of X irradiation; (**c**) Activity of a short *vegf-c* promoter (CR-MI); (**d**) Activity of an artificial promoter having three binding sites for NF-kB (CR-MI); (**e**) Activity of a VEGF-C 3′UTR reporter gene (CR-MI). * and *, significantly (*P*<0.05) increased promoter activity after P and X irradiation, respectively, as compared to CT; **#** and **#**, significantly decreased activity of MUT, as compared to WT *vegf-c* promoter after P and X irradiation, respectively; **§**, significantly decreased promoter activity after P, as compared to X irradiation. CT, control (non-irradiated cells); MUT, mutated, WT, wild type.

In CAL33 cells, VEGF-C protein levels increased in a dose-dependent manner following both P and X irradiation. Furthermore, they were significantly lower after P irradiation. Within the AR-SI setting (Figure 2a), VEGF-C protein levels were significantly increased after a low and high dose of irradiation with either P (*P*=0.038 and *P*=0.046, respectively) or X (*P*=0.0002 for both dose types). A significantly lower expression was observed after a high dose of P, as compared to X irradiation (by 59%, *P*=0.018). However, significantly increased levels were observed after a high versus low dose of X irradiation (3-fold increase, *P*=0.002).

The VEGF-C protein induction was also maintained at significantly increased levels in CAL33 cells of the CR-MI group (Figure 2b), after both low and high doses of P and X irradiation (*P*<0.001), with significantly decreased levels after high doses of P versus X irradiation (by 50%, *P*<0.001). In addition, there were significantly increased levels after high, as compared to low doses of X irradiation (*P*=0.002). These observations were confirmed in CAL27 cells within the CR-MI setting (Supplementary Figure S2B), where VEGF-C protein levels were significantly increased after both P and X irradiations (*P*<0.001), with lower levels after high doses of P versus X irradiation (*P*=0.001).

### X and P irradiations stimulate the VEGF-C promoter activity

Irradiation by either X or P stimulated the activity of the *vegf-c* promoter especially in CAL33 cells surviving to multiple X irradiations (6- and 18-fold increase, respectively, *P*<0.001, Figure 2c). This result is consistent with the induction of the VEGF-C mRNA within the CR-MI setting (Table 1) and suggests a chronic induction of *vegf-c* gene transcription, an increase in *vegf-c* mRNA half-life or a combination of both mechanisms. Mutation of the NF-κB binding site (MUT) had no effect on the basal *vegf-c* promoter activity in non-irradiated cells. However, in cells surviving to MI by P and X, the activity of the MUT, as compared to WT, promoter was significantly decreased (by 33%, *P*=0.004 and by 30%, *P*=0.027, respectively, Figure 2c) suggesting that the increase in the transcriptional activation of the *vegf-c* promoter depends in part on a constitutive activation of NF-κB. In the CAL27 cell line, the irradiation by either P or X did not stimulate the activity of the WT *vegf-c* promoter but the activity of the MUT promoter was completely inhibited in both non-irradiated and irradiated cells (*P*<0.001, Supplementary Figure S2C). To further assess the role of NF-κB on *vegf-c* promoter, the activity of an artificial promoter containing three binding sites for human NF-κB was determined in control and irradiated cells. In CAL33, the NF-κB-dependent promoter activity was lower in P irradiated cells, which is consistent with the activity of the *vegf-c* promoter having a WT NF-κB binding site (Figure 2d). For CAL27, the NF-κB-dependent promoter activity is almost equivalent in control and either X or P irradiated cells (Supplementary Figure S2D). This result indicates that the *vegf-c* promoter activity exclusively relies on an NF-κB-dependent transcriptional mechanism in CAL27 cells, whereas the dependency to NF-κB is partial in CAL33 cells. Moreover, a reporter gene used to assess VEGF-C mRNA half-life was not affected by either P or X irradiation in CAL33 cells (Figure 2e), suggesting that the increase in *vegf-c* mRNA levels does not depend on modifications in mRNA half-life.

### Cells surviving multiple irradiations by P and X generate tumors with distinct characteristics

**Figure 3:**
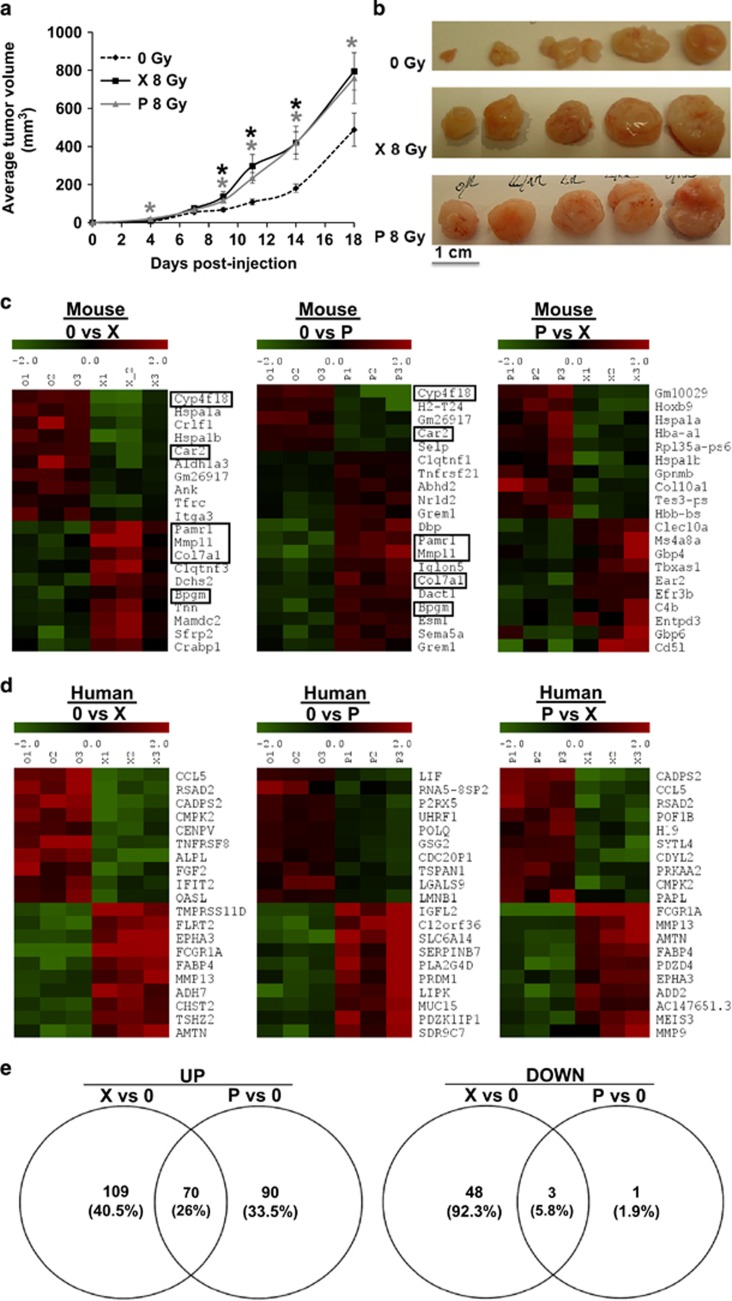
Evaluation of tumors generated following xenografting of either non-irradiated, P or X irradiated CAL33 cells in immunodeficient mice. (**a**) Average tumor volume (mm^3^); (**b**) Representative images of tumor xenografts; (**c**) Heatmap of 10 most up- and downregulated mouse genes in tumors generated by non-irradiated cells versus P or X tumors, and in P versus X tumors; (**d**) Heatmap of 10 most up- and downregulated human genes in tumors generated by non-irradiated cells versus P or X tumors, and in P versus X tumors; (**e**) Venn diagrams showing common upregulated and downregulated human genes between P and X tumors. Framed genes are commonly expressed in P and X tumors. Selection is adjusted *P*-value<0.05 and lofFC>1.

The cells resistant to MI by either P or X served to generate experimental tumors in mice to test their relative aggressiveness. The average tumor volume was significantly increased (*P*<0.05) for P and X tumors, but no differences were observed between the irradiation types (Figure 3a and b). These results were inconsistent with the *in vitro* proliferative abilities of the cells surviving after MI with either P or X. To determine whether P and X irradiated cells ‘educated’ the microenvironment to favor tumor growth, we performed a whole transcriptomic screening of the tumors. Indeed, distinct profiles for both the mouse (Figure 3c) and human (Figure 3d) 10 most up- and downregulated genes were detected. Among the 10 most up- and downregulated mouse genes, some (Figure 3c) such as collagen type XVII alpha 1 and carbonic anhydrase 2 (Car2)^26^ had a shared pattern of expression in P and X tumors (Figure 3c). In addition, we identified distinct profiles for the 10 most up- and downregulated mouse (Supplementary Figure S3) and human (Supplementary Figure S4) genes involved in angiogenesis, inflammation, metastasis, M1/M2 macrophage transition. Some of these genes had a shared pattern of expression in P and X tumors.

Furthermore, we identified 70 (26%) common upregulated and 3 (5.8%) common downregulated genes (Figure 3e) between X and P tumors, with roles in angiogenesis/metastasis, inflammation, M1/M2 macrophage transition and proliferation (Table 2).

**Figure 4:**
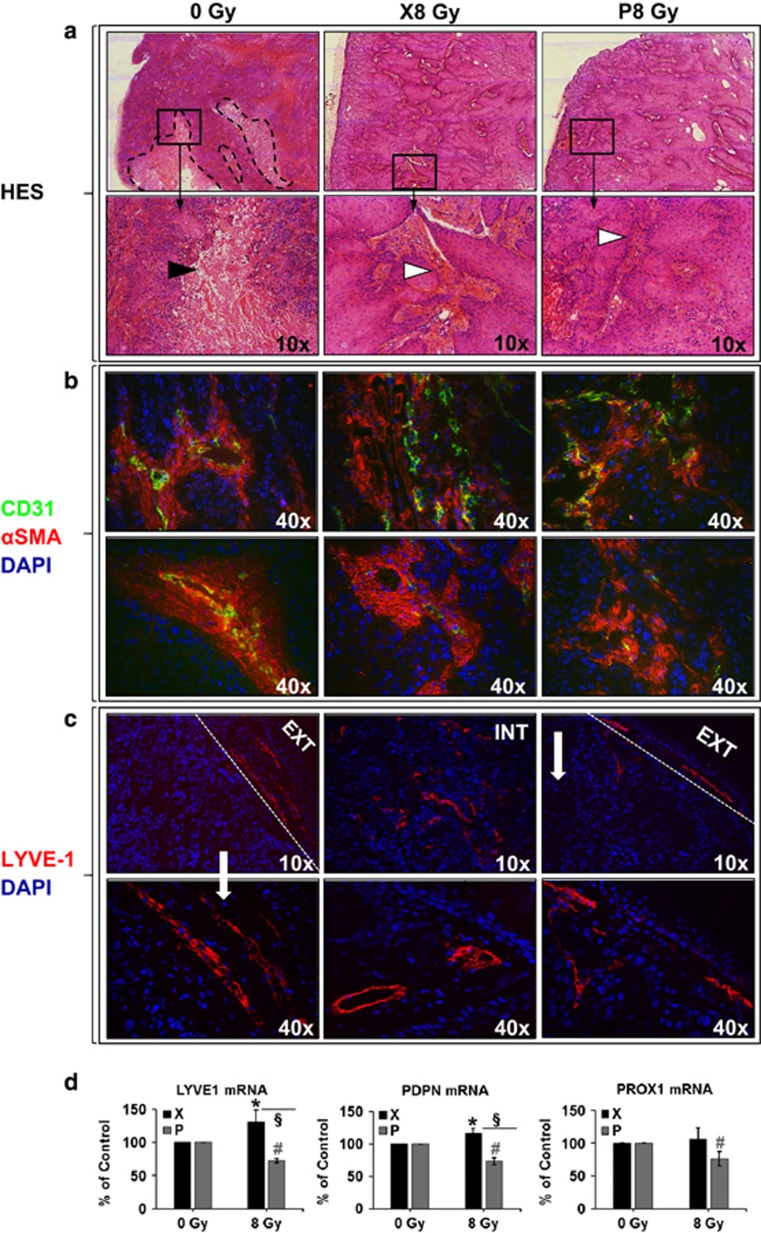
Histology, immunofluorescence and quantitative gene expression of vascular and lymphatic markers in murine xenografts. (**a**) Representative images of HES staining, indicating increased necrosis (black arrowhead, delimited by dashed black lines) in CT and increased blood vessels density (white arrowhead showing collagen surrounding the vessels) in the irradiated cells-derived tumors; (**b**) Representative images of CD31 (endothelial cells, green)/αSMA (pericytes, red)/Hoechst (nuclei, blue) staining, showing anarchic blood vessels structures and lack of pericyte coverage of blood vessels in the irradiated cells-derived tumors; (**c**) Representative images of LYVE1 (lymphatic endothelial cells, red)/Hoechst (nuclei, blue) staining, showing different patterns of lymphatic vessels development in X (both periphery and interior of the tumor), P and CT (periphery of the tumor) groups; dashed white lines delimit the tumor edge; CT, control (tumors generated by non-irradiated cells); (**d**) Murine LYVE1, PDPN and PROX1 mRNA quantitative mRNA expression, as percentage of control (0 Gy). HES, Hematoxylin Eosin Saffron.

Tumors induced by irradiated cells presented less necrosis and increased intra-tumor vessels density (*P*=0.031 for P and *P*=0.002 for X group, Figure 4a and Supplementary Figure S5A). In addition, irradiation by either P or X led to generation of tumors with destabilized vessel architecture (Figure 4b), attested by a decrease in vessels with co-staining for CD31 and αSMA (*P*<0.001 for both P and X groups, Supplementary Figure S5B). Lymphatic vessels were detected in the tumor-skin border of the control and P groups; however, they were also present in the core of the X tumors (Figure 4c), finding consistent with the over-expression of VEGF-C observed *in vitro*. Since VEGF-C was particularly discriminative between the two experimental irradiation conditions, we tested whether it had induced the development of lymphatic vessels. LYVE1, PDPN and PROX1 markers of lymphatic vessels were then tested. Density of LYVE1-positive lymphatic vessels was significantly increased in tumors generated with X-, as compared to non- and P-irradiated cells (*P*=0.006 and *P*=0.009, respectively, Supplementary Figure S5C). LYVE1 and PDPN mRNA were upregulated (*P*=0.015 and 0.044, respectively) in X and downregulated (*P*<0.001 for both markers) in P tumors. Lower mRNA levels of LYVE1 and PDPN were detected in P, as compared to X tumors (*P*=0.003 and *P*<0.001, respectively). PROX1 mRNA level was downregulated (*P*=0.02) and unchanged in P and X tumors, respectively (Figure 4d).

### Conventional radiotherapy by X increases tumor lymphangiogenesis in patients with HNSCC

**Figure 5:**
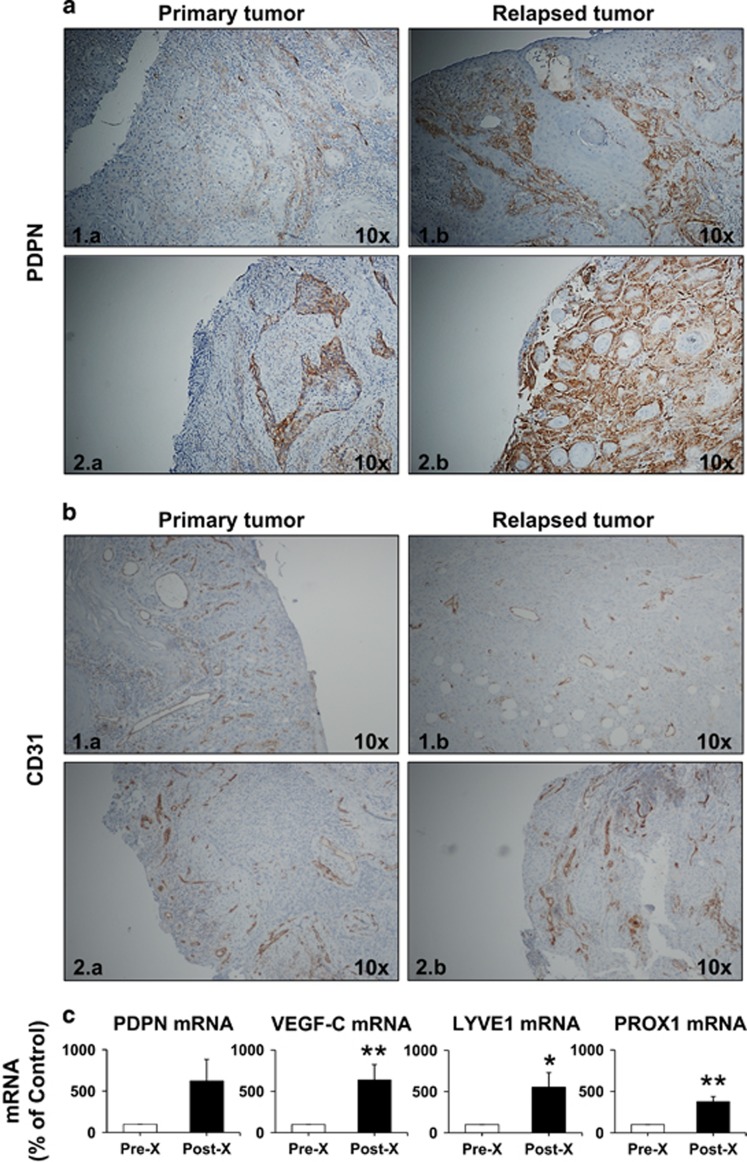
Evaluation of vascular and lymphatic markers in biopsies from patients diagnosed with HNSCC. Representative images of immunohistochemistry for (**a**) PDPN and (**b**) CD31 expression: (**1**) oral and (**2**) hypopharyngeal localization; Left panels (**1.a**, **2.a**)—primary tumor; Right panels (**1.b**, **2.b**)—relapsed tumor in the same patient after surgery and chemo-X radiotherapy (brown, PDPN/CD31; blue, hematoxylin - nuclei); (**c**) quantitative PDPN, VEGF-C, LYVE1 and PROX1 mRNA expression, as percentage of control (0 Gy); * and **, significantly increased values (*P*<0.05 and *P*<0.01, respectively) post-, as compared to pre-X radiotherapy.

To further correlate the relationship between irradiation-dependent VEGF-C expression and lymphatic vessels development, we tested the presence of lymphatic markers in biopsies from primary and locally relapsed human HNSCC, after conventional radiotherapy. Recent reports described that the expression of PDNP, one of the major makers of lymphatic vessels, was not restricted to lymphatic vessels but it was also expressed in HNSCC cells.^27^ Expression of PDPN was indeed detected in tumors from patients with oral and pharyngeal SCC (Figures 5a–1.a). However, we observed a significant increase of PDNP labeling, in both tumor and lymphatic cells, in sections from relapsed tumors after treatment with conventional X radiotherapy, as compared to the initial tumors (*P*=0.048, Figures 5a–1.b, Supplementary Figure S6A). In the same patients, the vascular network, attested by CD31 labeling, was not modified (*P*=0.059, Supplementary Figure S6B) in the relapsed (Figures 5b–1.b), as compared to the initial tumors (Figures 5b–1.a). In addition, a tendency for increased mRNA expression of PDPN (*P*=0.088), along with significantly increased mRNA expression of VEGF-C (*P*=0.005), LYVE1 (*P*=0.025) and PROX1 (*P*=0.003) were detected in relapsed patient tumors after conventional X radiotherapy (Figure 5c).

## Discussion

Our *in vitro* results indicate that P irradiation led to lower expression of factors involved in (lymph)angiogenesis, inflammation and immune tolerance. This suggests the acquisition of less aggressive phenotypes after P therapy. The selection of surviving cells was still possible after MI, indicating a mechanism of acquired resistance secondary to irradiation.^28^ However, the molecular profiling of the surviving cells suggests a more aggressive *in vivo* phenotype after MI with X. Therefore, due to its physical and biological properties, P irradiation may be more efficient in tumor size control through dose escalation.

The long-term surviving cells after three irradiations with P showed a downregulation of the investigated pro-angiogenic/pro-inflammatory genes, except for *vegf-c*, while most of these genes were upregulated after X irradiation. The implication of VEGF-C in the metastatic dissemination process after irradiation has not been elucidated. To our knowledge, this is the first report showing P or X radiation-induced VEGF-C over-expression at both gene and protein levels in HNSCC cells. The VEGF-C mRNA levels increased in a dose-dependent manner and with the irradiation number, except in the cells surviving after three irradiations with P. These observations suggest that P radiotherapy would lead to less pronounced lymphangiogenesis/metastasis, as compared to X radiotherapy.

Therefore, we postulated that over-expression of VEGF-C may represent an extrinsic mechanism responsible for the post-irradiation tumor dissemination/metastasis in HNSCC. VEGF-C expression was associated with lymph node metastasis, recurrence and a poorer five-year survival rate in patients with HNSCC,^11^ being an independent prognostic factor.^11, 29^ Moreover, the online available database cBioPortal (http://www.cbioportal.org) shows that over-expression of VEGF-C correlated to significantly lower disease-free (*P*=0.0022, Supplementary Figure S7A) and overall (*P*=0.015, Supplementary Figure S7B) survival rates in patients with HNSCC (*n*=517). It has been reported that gamma rays irradiation induced VEGF-C expression and endothelial cell proliferation in lung cancer.^30^ These observations, corroborated with ours, suggest that VEGF-C may be an important therapeutic target for HNSCC patients who relapse after radiotherapy with either P or X.

Because VEGF-C might be a major factor responsible for post-irradiation disease progression in HNSCC patients, via promotion of lymphangiogenesis, we further started investigating the mechanisms involved in its induction, which may serve to its therapeutic targeting. Regulation of VEGF-C expression has been poorly addressed.^27, 31, 32^ Irradiation-mediated induction of VEGF-C mRNA suggested stimulation of transcription, stabilization of mRNA or a combination of these mechanisms.^31^ Our data indicate that both P and X irradiation stimulated the activity of a short form of *vegf-c* promoter in CAL33 cells. The *vegf-c* promoter contained a binding site for NF-κB. The dependency of this site is variable considering the two cell lines we tested, but nevertheless NF-κB plays a key role in VEGF-C regulation, as suggested in another cancer type.^32^ As these cell lines came from two different patients, our results highlight the inter-patient variability in VEGF-C expression and regulation, stressing out the importance of implementing personalized diagnosis and treatment strategies.

In the cells surviving after three irradiations, the VEGF-A and VEGF-D genes were downregulated by P and upregulated by X irradiation. VEGF-A expression significantly correlated with lymph node metastasis in patients with HNSCC.^11^ High VEGF-A expression was also associated with higher clinical stages and worse overall survival, being a significant predictor of poor prognosis in patients with HNSCC.^33^ Furthermore, VEGF-D expression correlated with lymphatic vessel density and lymph node metastasis in these patients.^10^ In addition, VEGFR-2, VEGFR-3 and NRP1, highly expressed by HNSCC cells,^34^ were downregulated in the surviving cells selected after three irradiations with P, but not with X. High NRP1 and NRP2 levels correlated with poor prognosis in HNSCC patients, NRP2 being an independent prognostic markers for overall survival.^35^

Therefore, our study sets the basis for clinical assays investigating more efficient treatments, combining P radiotherapy with anti-angiogenic-targeted therapies. Such combinations would eventually lead to decreased selection of post-irradiation surviving cells and lower relapse rates in patients with HNSCC, for which the current treatments include X irradiation.^3^ A case report describing the successful treatment of a patient with chondrosarcoma by combining P radiotherapy with sunitinib, an inhibitor of VEGFRs and platelet-derived growth factor receptor, underlines the effectiveness of such approach.^36^

We also showed that P and X radiations differently modulated the pro-inflammatory gene expression in HNSCC cells. Among the assessed genes, the highest determined mRNA level was for IL-8. Stress and drug-induced IL-8 signaling conferred chemotherapeutic resistance to cancer cells.^37^ Serum and tumor IL-8 significantly affected the disease-free survival in patients with early stage HNSCC.^38^ Therefore, inhibiting the effects of IL-8 signaling in combination to chemoradiotherapy may be of significant therapeutic value.

P but not X irradiation downregulate IL-6 expression at the mRNA level. IL-6 expression predicted a poor response to radio-chemotherapy and a non-favorable prognosis in HNSCC patients.^39^ It was also linked to radiation resistance and development of chronic toxicities after irradiation.^40^ Depending on tumor location, the most common side effects after conventional radiotherapy of HNSCC include mucositis, xerostomia, dysphagia requiring short-term or permanent gastrostomy, soft tissue/bone necrosis, neck fibrosis, and thyroid dysfunction.^41^ Although the primary goal in radiotherapy is tumor control, a parallel essential goal is to spare normal tissues from radiation toxicity. Therefore, our data bring further pre-clinical evidence that the use of P irradiation in the treatment of HNSCC may lead to less inflammatory side effects.

We also showed that, in the cells surviving long-term after three irradiations, another major pro-inflammatory cytokine, CCL2, was downregulated after P, while being highly upregulated after X irradiation. As serum CCL2 levels were associated with HNSCC progression,^42^ our data suggest that P therapy might be more beneficial for these patients.

Our results also showed that PLK1 and TRF2 genes were differently regulated after P or X irradiation and correlated to the proliferation patterns. By inhibiting apoptosis, PLK1 over-expression was associated with poor survival in patients with HNSCC, being an independent prognostic factor.^43^ Its targeting with a multi-kinase inhibitor led to encouraging anti-tumor activity in patients with SCC.^44^ These data suggest that PLK1 might be a potential therapeutic target for HNSCC patients undergoing radiotherapy. TRF2 may also become an established predictive marker for treatment efficacy and a marker of survival in HNSCC. We previously showed that the treatment response was increased in TRF2 knocked-down cells and that TRF2 over-expression had a negative impact on patients’ survival.^23^

Irradiation leads to adaptive changes in the tumor microenvironment that may limit the generation of an anti-tumor immune response.^24^ Indeed, we showed a significant increase of PD-L1 expression after P, and confirmed the X radiation-induced PD-L1 expression in other cancers.^24, 45^ In patients with HNSCC, high PD-L1 expression in primary tumors correlated with metastasis and poor prognosis, being an independent prognostic factor.^46^ PD-L1 was also a significant predictor for poor treatment response and shorter survival in X radiotherapy-treated patients with HNSCC.^45^ A phase II, multi-center, single-arm, global study of monotherapy with durvalumab, a Fc optimized monoclonal antibody directed against PD-L1, is ongoing in our institution in patients with recurrent/metastatic HNSCC and PD-L1 positive status. Therefore, our data, associated to the progress in the field, set the basis for the investigation of novel therapeutic strategies for HNSCC, based on the PD-L1–PD-1 interaction, in combination with radiotherapy.

We also demonstrated that the aggressiveness of the irradiated cells was augmented *in vivo* through increased tumor volume, density of tumor vessels and blood vessels with destabilized architecture. These observations suggest that the irradiation-adapted cells have acquired different transcriptome and secretome profiles. Indeed, among the common human genes upregulated in either X or P tumors, but downregulated in tumors generated with non-irradiated cells, we identified PDZK1 interacting protein 1 (PDZK1IP1, known also as MAP17)^47^ and fibronectin leucine rich transmembrane protein 2,^48^ known for promoting cell proliferation. In addition, mouse Car2 expression was downregulated in P and X tumors, while upregulated in tumors generated with non-irradiated cells. Interestingly, low CAR2 protein expression has been associated with increased tumor size.^26^ In addition, the X tumors showed upregulation of human genes involved in metastasis, angiogenesis and epithelial mesenchymal transition, such as MMP2, MMP9, MMP13, MMP16, MMP28 and vimentin,^15^ while P tumors showed upregulation of human C–C Motif Chemokine Ligand 5 chemokine gene involved in CD8+ T lymphocytes recruitment associated with better clinical outcomes.^49^

To get further insights whether tumor cell adaptation following radiotherapy may contribute to clinical disease progression, in part through lymphangiogenesis, we investigated lymphatic markers expression in patients with relapsed HNSCC after X radiotherapy. Biopsies at relapse are very rarely sampled in radiotherapy-treated patients. However, in this small cohort, all patients presented increased protein and/or mRNA levels of PDPN, VEGF-C, LYVE1 and PROX1, bringing evidence that conventional radiotherapy may promote lymphangiogenesis. It has also been reported by others that high PDPN expression is associated with aggressive tumor behavior, poor prognosis and metastatic regulation through interaction with VEGF-C, suggesting that PDPN may be used as a potential prognostic biomarker for HNSCC.^27^ However, our *in vitro* studies did not reveal increased PDPN expression in HNSCC cells that resisted to MI (Supplementary Figure S8).

In conclusion, our study highlighted the differential gene/protein expression profile after P versus X irradiation in HNSCC and potential candidate markers for prognosis, efficacy of anti-tumor treatments and new anti-tumor targets, such as VEGF-C. Beside the physical advantage of P irradiation in dose deposition, our observations provide preclinical evidence that beam therapy with P might be superior to conventional X therapy in HNSCC patients, due to its biological advantages. P irradiation could therefore permit dose escalation without increasing the side effects, while increasing the tumor control. Further work is also needed to refine the strategies for blocking VEGF-C activity and its effects on the vascular/lymphatic endothelial or tumor cells with anti-angiogenic therapies. The implementation of P therapy in combination with anti-angiogenic or anti-immune checkpoint drugs for HNSCC will therefore require prospective randomized clinical trials to measure the toxicity and disease control.

## Materials and methods

### Cell lines and culture

Two human HNSCC cell lines, CAL33 and CAL27, were provided through a Material Transfer Agreement with the Oncopharmacology Laboratory, Centre Antoine Lacassagne (CAL), where they had initially been isolated.^50^ The cells were cultured in Dulbecco’s modified Eagle's medium supplemented with 7% fetal bovine serum (Thermo Fisher Scientific, Waltham, MA, USA).

### Cell irradiations

Five million cells were seeded onto 12 cm^2^ tissue culture flasks, 48 h prior to the irradiations, which were carried out at CAL (four independent experiments) with either P (63 MeV Cyclotron MEDICYC, CAL, Nice, France) or X (6 MeV Dual energy Clinac 21EX Linear Accelerator, Varian Inc., Palo Alto, CA, USA). For clonogenicity assays, the cells were irradiated once (single irradiation, SI) with 1, 2, 4, 6 or 8 Grays (Gy; physical dose) and processed immediately after irradiation. To the purpose of all other experiments, the cells were irradiated either once or three times, 1 week apart (multiple irradiations, MI) with either 2 Gy (low dose) or 8 Gy (high dose), and processed 6 h after irradiation. In the MI setting, cells were re-seeded after each irradiation and kept in culture until the next irradiation to reproduce the clinical situation where patients are usually given several irradiations. The CR was evaluated to determine if the changes associated with the AR persist late (3 weeks) after irradiation.

Two cell groups were thus generated from each independent irradiation experiment. They consisted of cells subjected to: (1) SI and analysis 48 h thereafter (AR-SI); (2) MI and culture expansion (3 weeks) after the third irradiation (CR-MI). All cell experiments were performed in triplicate wells for each condition and repeated at least three times.

### Clonogenicity assays

They were performed to quantify the radio-induced cell mortality, to generate the cell surviving curves and to determine the RBE. Owing to radiation dose-induced differences in plating efficiency, the cells were seeded at different densities: 3000 cells/dish for 0, 1, 2 and 4 Gy; 6000 cells/dish for 6 Gy and 9000 cells/dish for 8 Gy. On day 10 of culture, cells were stained for 20 min with Giemsa (Sigma Aldrich, St. Louis, MO, USA). Stained plates were scanned and the number of cell colonies was determined with the ImageJ processing software (National Institutes of Health, Bethesda, MD, USA). The RBE was calculated as ratio of the biological effectiveness of P versus X irradiation, given the same dose/amount of absorbed energy.^25^

### Cell counting for viability and proliferation assessment

The cell counting for the CR-MI group was done every day, for 4 days post-seeding, with an automatic cell counter (Advanced Detection Accurate Measurement system, LabTech, Tampa, FL, USA), according to the manufacturer’s instructions.

### Quantification of gene expression

Molecular characterization of the irradiated cells was done by using the quantitative real-time–polymerase chain reaction. Total RNA was extracted with the RNeasy Mini Kit; first-strand cDNA synthesis was performed by using the QuantiTect Reverse Transcription Kit (all from Qiagen, Hilden, Germany). cDNA samples were amplified by using the StepOnePlus RT–PCR System (Thermo Fisher Scientific) for 40 cycles with the Takyon Rox SYBR Master Mix, dTTP Blue (Eurogentec, Liege, Belgium) and specific oligonucleotides (Sigma Aldrich, Supplementary Table S1), to assess mRNA expression for VEGF-A, VEGF-C, VEGF-D, VEGFR-1, VEGFR-2, VEGFR-3, NRP1, NRP2, IL-6, IL-8, CCL2, TRF2, PLK1, PD-L1, LYVE1, PDPN and PROX1. mRNA levels were normalized to a housekeeping mRNA coding for either the human or murine ribosomal protein, large, P0 (RPLP0). The gene expression levels were given the individual scores of −1, 0 and 1 when they were significantly decreased, not significantly changed and significantly increased, respectively, as compared to control. For each irradiation setting, a global gene expression score was then calculated by cumulating the individual scores allocated to each gene expression level.

### Protein quantification

VEGF-C protein was quantified by using an enzyme-linked immunosorbent assay (human DuoSet ELISA kit, R&D Systems, Minneapolis, MN, USA). Protein concentration was normalized to the viable cell number.

### Luciferase assays

CAL33 cells belonging to the CR-MI group were transfected by using 50 μl NaCl buffer, 1.25 μl of polyethylenimine transfection reagent (Sigma Aldrich) and 0.5 μg of total test plasmid DNA-renilla luciferase. The plasmids encoded either (i) a human *vegf-c* promoter fragment with either a non-mutated (wild type, WT) or a mutated (MUT) binding site for the nuclear factor kappa-light-chain-enhancer of activated B cells (NF-κB),^32^ (ii) an artificial promoter containing three binding sites for human NF-κB or (iii) a human VEGF-C 3′UTR reporter (LightSwitch, S803537, Active Motif, Carlsbad, CA, USA), all cloned downstream of the luciferase reporter gene. A CMV plasmid was used to control the variability of transfection efficiency in the reporter assays.

### Tumor xenografts

The study was carried out in strict accordance with the recommendations of the United Kingdom Coordinating Committee on Cancer Prevention Research’s Guidelines for the Welfare of Animals in Experimental Neoplasia. Our experiments were approved by the ‘Comité National Institutionnel d'Éthique Pour l'Animal de Laboratoire’ (CIEPAL, reference: NCE/2013-97). One million non-irradiated, P or X irradiated CAL33 cells (CR-MI group) were injected subcutaneously into the flank of 6-week-old NMRI-Foxn1^nu^/Foxn1^nu^ female mice (Janvier Labs, Le Genest-Saint-Isle, France, *n*=10/group). The tumor volume (v=L × l^2^ × 0.52) was determined following measurement with a caliper. When the tumors reached 1 cm^3^, the mice were killed and the tumors collected.

### Whole transcriptomic screening of tumor xenografts

For the sequencing and secondary analysis, 1 μg of total RNA was extracted from tumor xenografts, generated with either non-irradiated, P or X irradiated cells (*n*=3/group), by using the AllPrep DNA/RNA/Protein Mini Kit (Qiagen). Lack of RNA degradation (ratio 28S/18S⩾1.6 and RIN>7) was documented (Bioanalyzer 2100, Agilent Technologies, Santa Clara, CA, USA). The libraries were generated by using Truseq Stranded mRNA kit (Illumina, San Diego, CA, USA). Libraries were then quantified with KAPA library quantification kit (Kapa Biosystems, Inc., Wilmington, MA, USA) and pooled; 4 nM of this pool were loaded on a Nextseq 500 high output flowcell and sequenced with a 2 × 75 bp paired-end chemistry. STAR (2.4.0i) was used to map reads versus a STAR database containing: Ensembl hg19 build (GRCh37.75), Ensembl mm10 build (GRCm38) and the ERCC spikes-in set, formatted with splice junctions information described from Ensembl release GRCh37.75 and GRCm38.83. STAR options were set to the recommended Encode RNA-seq options ‘—outFilterType BySJout —outFilterMultimapNmax 20 —alignSJoverhangMin 8 —alignSJDBoverhangMin 1 —outFilterMismatchNmax 999 —outFilterMismatchNoverLmax 0.04 —alignIntronMin 20 —alignIntronMax 1000000 —alignMatesGapMax 1000000’. Gene counts were obtained with featureCounts (subread-1.5.0-p3-Linux-x86_64) and ‘—primary -p -s 1 -C’ options, by using the same GTF files used for STAR splice junctions training. Data were deposited in Gene Expression Omnibus (accession code GSE90761, https://www.ncbi.nlm.nih.gov/geo/query/acc.cgi?token=opybisygbzotvkh&acc=GSE90761).

For the heatmaps gene lists selection, genes involved in angiogenesis, inflammation, metastasis and cell proliferation were selected by using the Ingenuity Pathway Analysis (Qiagen) database. To define M1/M2 macrophages-related genes, the GEO data set GSE69607 has been reanalyzed by using geo2R online resource. Genes up- and downregulated (Abs (logFC)>2) in both M1 versus M0 and M2 versus M0 comparisons were selected as the ‘M1/M2 macrophages’-related gene list.

### Histochemistry and immunofluorescence

Murine tumor sections were handled as previously described.^8^ To assess tumor architecture, the sections were subjected to hematoxylin eosin saffron staining. For immunofluorescence, the frozen sections were incubated overnight, at 4 °C, with the following primary antibodies: polyclonal rabbit anti-mouse/human LYVE1 (1:200; Abcam, Cambridge, UK), monoclonal mouse anti-mouse/human alpha smooth muscle actin (αSMA, 1:400, Sigma Aldrich) and monoclonal rat anti-mouse CD31 (1:50, clone MEC 13.3, BD Pharmigen, Heidelberg, Germany) primary antibodies, then incubated for 2 h at room temperature, in the dark, with the secondary antibodies: anti-rabbit FP594, anti-mouse FP547 (1:1000, FluoroProbes, Interchim, Montluçon, France) and anti-rat AF488 (1:1000, AlexaFluor, Thermo Fisher Scientific); cell nuclei were stained with Hoechst (1:1000, Thermo Fisher Scientific). Cell and tissue preparations were examined under an inverted epifluorescence microscope (Axio Observer Z1) with an incorporated digital camera system for imaging (AxioCam Icc1); images acquisition and stitching, as well as the assessment of tumor vessels density, were performed with ZEN 2.3 software (all from Carl Zeiss MicroImaging GmbH, Weinheim, Germany).

### Immunohistochemistry

Patient biopsy samples were collected with the approval of the local Ethics Committee, and their use in research was in accordance with the Declaration of Helsinki. The patient, disease and treatment characteristics were described in Supplementary Table S2. Sections from formalin-fixed and paraffin-embedded biopsies from initial and relapsed tumors were incubated at room temperature with monoclonal, primary mouse anti-human PDPN and CD31 antibodies, as well as biotinylated secondary antibodies, by using an automated slide stainer (Ventana Medical Systems, Inc., Basel, Switzerland). Binding was detected with the diaminobenzidine substrate against a hematoxylin counterstain. Evaluation of marker expression was performed by an accredited clinical pathologist (IP).

### Statistical analyses

Statistical analysis for all test, excepting whole transcriptomic screening, was performed by two-tailed unpaired *t* test on at least three independent experiments; the results were considered statistically significant when *P*-value<0.05. The error bars were defined as standard error of the mean. For the whole transcriptomic screening, statistical analyses were conducted separately for human and mouse gene expression counts. Quality of libraries was assessed based on the Pearson correlation between observed versus expected ERCC counts (*R*^2^>0.90 for all samples). Normalization and differential analysis were conducted within R/Bioconductor environment, by using DESeq2. *P*-values were corrected for multiple testing, by using the Benjamini and Hochberg method. Heatmaps were generated with TMeV software. Heatmaps used the top 10 most up- and downregulated genes, based on logFC and adjusted *P*-value<0.05 for human genes, and logFC only for mouse genes.

## Figures and Tables

**Table 1:** Quantitative gene expression, as percentage of control (0 Gy), in either P or X irradiated CAL33 cells belonging to (a) AR-SI and (b) CR-MI groups

*(a) mRNA levels (% of control)−AR−SI*		*X*		*P*	
*Role*	*Gene*	*2 Gy*	*8 Gy*	*2 Gy*	*8 Gy*
(Lymph)angiogenesis and metastasis	VEGF-A	132	a191	143	160
	VEGF-C	130	a227	159	a209
	VEGF-D	103	96	105	98
					
Inflammation	IL6	129	136	119	a, b72
	IL8	147	a436	162	a401
	CCL2	104	123	b79	a136
					
Proliferation	TRF-2	88	a112	93	102
	Plk-1	85	a108	89	a99
					
Anti-tumor immunity	PD-L1	108	103	91	112
					
Gene score		4	6	0	3

*(b) mRNA levels (% of control)–CR–MI*		*X*		*P*	
*Role*	*Gene*	*2 Gy*	*8 Gy*	*2 Gy*	*8 Gy*
(Lymph)angiogenesis and metastasis	VEGF-A	118	a208	b93	a, b107
	VEGF-C	281	a626	226	b215
	VEGF-D	127	134	b95	b79
	VEGFR-1	96	a73	b72	73
	VEGFR-2	81	88	76	77
	VEGFR-3	116	91	100	80
	NRP1	105	90	b92	85
	NRP2	133	a169	b102	b129
					
Inflammation	IL6	120	126	b74	a, b100
	IL8	87	a7916	b75	b90
	CCL2	112	a751	b83	b94
					
Proliferation	TRF-2	101	95	95	106
	Plk-1	91	97	89	a117
					
Anti-tumor immunity	PD-L1	119	140	b92	a116
					
Gene score		4	6	−8	−4

Highlighted values—significantly different (*P*<0.05) expression levels, as compared to control, for genes associated to favorable (dark gray) and non-favorable (black) outcomes.

a significantly different expression levels after low, as compared to high dose(s) of either P or X irradiation.

b significantly different expression levels after either low or high dose(s) of P, as compared to X irradiation.

**Table 2:** Common upregulated and downregulated human genes in tumors generated with either X or P irradiated cells

*Common up-regulated genes between X* versus *0 and P* versus *0*		
*Role*	*Gene abbreviation*	*Gene full name*
Metastasis/Angiogenesis		
	KRT16	Keratin 16
	SERPINB3	Serpin family B member 3
	CAPNS2	Calpain small subunit 2
	GRHL3	Grainyhead like transcription factor 3
	CSTB	Cystatin B
	PRSS27	Protease, serine 27
	TLE4	Transducin like enhancer of split 4
	TMPRSS11D	Transmembrane protease, serine 11D
		
Inflammation	PGLYRP3	Peptidoglycan recognition protein 3
	RASGRP1	RAS guanyl releasing protein 1
	ENDOU	Endonuclease, poly(U) specific
	METRNL	Meteorin like, glial cell differentiation regulator
	S100A8	S100 calcium binding protein A8
	S100A9	S100 calcium binding protein A9
	A2ML1	Alpha-2-macroglobulin like 1
	HCN2	Hyperpolarization activated cyclic nucleotide gated potassium channel 2
	CHST2	Carbohydrate sulfotransferase 2
		
M1/M2	ABCG1	ATP binding cassette subfamily G member 1
		
Proliferation	HPGD	Hydroxyprostaglandin dehydrogenase 15-(NAD)
	BNIPL	BCL2 interacting protein like
	PPP2R2C	Protein phosphatase 2 regulatory subunit Bgamma
	KLK8	Kallikrein related peptidase 8
	GJB6	Gap junction protein beta 6
	EEF1A2	Eukaryotic translation elongation factor 1 alpha 2
	EPHA4	EPH receptor A4
	GAS7	Growth arrest specific 7
	DSG1	Desmoglein 1
	**PDZK1IP1**	PDZK1 interacting protein 1
	TMPRSS11A	Transmembrane protease, serine 11A
	**FLRT2**	Fibronectin leucine rich transmembrane protein 2
		
Other	C12orf36	Putative uncharacterized protein C12orf36
	FRMPD1	FERM and PDZ domain containing 1
	TMEM45A	Transmembrane protein 45A
	LIPK	Lipase family member K
	CTC-490G23.2	CTC-490G23.2
	HOPX	HOP homeobox
	PLIN2	Perilipin 2
	SDR9C7	Short chain dehydrogenase/reductase family 9C, member 7
	STXBP5-AS1	STXBP5 antisense RNA 1
	ARRDC4	Arrestin domain containing 4
	FRY	FRY microtubule binding protein
	FAM25A	Family with sequence similarity 25 member A [
	SCEL	Sciellin
	GJB2	Gap junction protein beta 2
	UNC5B-AS1	UNC5B antisense RNA 1
	RP11-21B23.2	Pre-mRNA processing factor
	SPRR1B	Small proline rich protein 1B
	NAV3	Neuron navigator 3
	SLC10A6	Solute carrier family 10 member 6
	RP11-275I14.4	Pre-mRNA processing factor
	RP11-356I2.4	Pre-mRNA processing factor
	C9orf169	Cysteine rich tail 1
	RP11-321G12.1	Pre-mRNA processing factor
	LINC01094	Long intergenic non-protein coding RNA 1094
	OR7E62P	Olfactory receptor family 7 subfamily E member 62 pseudogene
	FAM3D	Family with sequence similarity 3 member D
	SMIM5	Small integral membrane protein 5
	FBXL16	F-box and leucine rich repeat protein 16
	RP11-783K16.5	Pre-mRNA processing factor
	KCNK7	Potassium two pore domain channel subfamily K member 7
	FAM25HP	Family with sequence similarity 25, member H pseudogene
	WI2-85898F10.1	Uncharacterized LOC107985535
	IVL	Involucrin
	TCN1	Transcobalamin 1
	KLHL4	Kelch like family member 4
	LRRC7	Leucine rich repeat containing 7
	RP11-557H15.3	Pre-mRNA processing factor
	TSHZ2	Teashirt zinc finger homeobox 2
	OLFM2	Olfactomedin 2
	ADH7	Alcohol dehydrogenase 7 (class IV), mu or sigma polypeptide

*Common down-regulated genes between X* versus *0 and P* versus *0*		
*Role*	*Gene abbreviation*	*Gene full name*
Proliferation	LIF	leukemia inhibitory factor
Other	RNA5-8SP2	RNA, 5.8S ribosomal pseudogene 2
	P2RX5	purinergic receptor P2X 5

In bold are shown genes upregulated in either P or X tumors, but down-regulated in tumors generated with non-irradiated cells. Selection is adjusted *P*-value<0.05 and lofFC>1.
