# Supplementary material for: Effects of proton versus photon irradiation on (lymph)angiogenic, inflammatory, proliferative and anti-tumor immune responses in head and neck squamous cell carcinoma
Source: Oncogenesis. 2017 Jul 3;6(7):e354–. doi: 10.1038/oncsis.2017.56 (PMC5541708; doi:10.1038/oncsis.2017.56)
Supplement: Supplementary Materials [file oncsis201756x1.docx]

**Legends of supplementary materials**

**Supplementary Table Legends**

**Supplementary Table S1. Sequences of the oligonucleotide primers used for qRT-PCR.**

**Supplementary Table S2. Diagnosis, disease and treatment characteristics of patients with HNSCC.**

**Supplementary Table S3. Quantitative gene expression, as percentage of control (0 Gy), in either P or X irradiated CAL27 cells belonging to CR-MI group.** Highlighted values – significantly different (p<0.05) expression levels, as compared to control, for genes associated to anti-tumor (dark grey) and pro-tumor (black) effects; *, significantly different expression levels after 2 Gy, as compared to 8 Gy of either P or X irradiation; **#**, significantly different expression levels after either 2 Gy or 8 Gy of P, as compared to X irradiation.

**Supplementary Figure Legends**

**Supplementary Fig. S1.** **Clonogenic survival of CAL33 cells after irradiation with escalating doses of either P or X**, indicating a Relative Biological Effectiveness of 1.1 in favor of P.

**Supplementary Fig. S2. (A) Proliferation curves, (B) VEGF-C protein expression levels, activity of a (C) VEGF-C promoter and (D) artificial promotor having three NF-κB binding sites, in CAL27 cells following high doses of either P or X irradiation (CR-MI setting).** (**A**) **#**, (p<0.05) and ## (p<0.01) significantly decreased cell counts at 72h and 96h, respectively, post-irradiation with high doses of P or X, as compared to CT; (**B**) * and *, significantly increased VEGF-C protein levels after high doses of P and X irradiation, respectively, as compared to CT; **#**, significantly decreased levels after high doses of P, as compared to X irradiation; (**C**) **#**, significantly decreased activity in CT, P and X irradiated cells transfected with a VEGF-C promotor with a MUT NF-κB binding site, as compared to CT cells transfected with a VEGF-C promoter having a WT NF-κB binding site; **§**, significantly decreased activity in cells transfected with a VEGF-C promoter having a MUT NF-κB binding site, as compared to the corresponding cells transfected with a VEGF-C promoter having a WT NF-Κb binding site; (**D**) Lack of stimulation of NF-κB promoter activity in irradiated cells; **#**, significantly decreased promoter activity in X irradiated, as compared to CT cells; CT, control (non-irradiated cells).

**Supplementary Fig. S3. Heatmap of ten most up- and down-regulated mouse genes involved in angiogenesis, inflammation, metastasis, M1/M2 macrophage transition and proliferation in tumors** generated by non-irradiated cells vs P or X tumors, and in P vs X tumors. Framed genes are commonly expressed in P and X tumors. Selection is Abs(logFC) > 1.

**Supplementary Fig. S4. Heatmap of ten most up- and down-regulated human genes involved in angiogenesis, inflammation, metastasis, M1/M2 macrophage transition and proliferation in tumors** generated by non-irradiated cells vs P or X tumors, and in P vs X tumors. Framed genes are commonly expressed in P and X tumors. Written in red are genes associated with disease progression; written in green are genes associated with favorable outcomes. Selection is adjusted p value < 0.05 and Abs(logFC) > 1.

**Supplementary Fig. S5. Density of (A) tumor vessels, (B) tumor blood vessels with CD31/αSMA colocalization and (C) lymphatic vessels with LYVE1 labeling.** * and *, significantly increased density in P and X tumors, respectively, as compared to CT; **#** and **#**, significantly decreased density in P and X tumors, respectively, as compared to CT; **§**, significantly decreased density in P, as compared to X tumors; CT, control (non-irradiated cells).

**Supplementary Fig. S6.** **Quantification of PDPN (A) and CD31 (B) protein expression in immunohistochemistry specimens from primary and relapsed tumors of HNSCC patients.** Results are reported as percentage of control (primary tumor); *, significantly increased values (p<0.05) in the relapsed tumors after surgery and chemo-X radiotherapy, as compared to the primary tumors.

**Supplementary Fig. S7. cBioPortal data** showing the correlation between VEGF-C over-expression and significantly lower (**A**) disease free and (**B**) overall survival rates in patients with HNSCC.

**Supplementary Fig. S8. Quantification of hPDPN mRNA expression in long-term surviving CAL33 cells**, selected *in vitro* after the third irradiation with either P or X (CR-MI setting).
